# Axillary lymph‐node metastases as the primary presentation of high‐grade serous ovarian carcinoma: A case report

**DOI:** 10.1002/ccr3.5724

**Published:** 2022-04-14

**Authors:** Monire Mirzaei, Abbas Eshraghi, Mahdiieh Ghoddoosi, Maedeh Alsadat Fatemi, Azhar Eshraghi, Sara Shenavaei, Danial Fazilat‐panah

**Affiliations:** ^1^ Clinical Research Development Center Nekouei‐Forghani Hospital Qom University of Medical Sciences Qom Iran; ^2^ Clinical Research Development Center Qom University of Medical Sciences Qom Iran; ^3^ Department of Pathology Shahid Beheshti Hospital Qom University of Medical Sciences Qom Iran; ^4^ Cancer Research Center Babol University of Medical Sciences Babol Iran; ^5^ Qom University of Medical Sciences Qom Iran; ^6^ 37552 Student Research Committee Mashhad University of Medical Sciences Mashhad Iran

**Keywords:** axillary lymph‐node metastases, carcinoma of unknown primary, high‐grade serous ovarian carcinoma, cancer of unknown primary

## Abstract

We reported a female presented with an initial diagnosis of metastatic axillary lymph‐node carcinoma that comprehensive assessments revealed a definitive diagnosis of high‐grade serous ovarian carcinoma as the primary tumor.

## Graphical abstract

In the absence of malignant lesion in a breast of a female patient with CUP limited to axillary lymph‐nodes, other potential sources of primary disease such as gastrointestinal tract and reproductive system should be investigated appropriately.

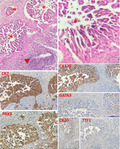

## 1 INTRODUCTION

Ovarian cancer is among the most common gynecological neoplasms.^1^ Its diagnosis is challenging due to the non‐specific presenting symptoms leading to a late diagnosis which is at the FIGO stage II‐IV in the most cases.^2^, ^3^ Despite the possibility of regional nodal involvement and value of surgical staging,^4^, ^5^ presence of distant lymph‐node metastasis is not a common event at the presentation, especially as the only site of distant involvement.^6^, ^7^ There are some reports on inguinal or axillary lymph‐node metastasis in these patients.^8^, ^9^ We reported a female presented with initial diagnosis of metastatic axillary lymph‐node carcinoma that comprehensive assessment revealed a definitive diagnosis of high‐grade serous ovarian carcinoma.

## 2 CASE PRESENTATION

**FIGURE 1:**
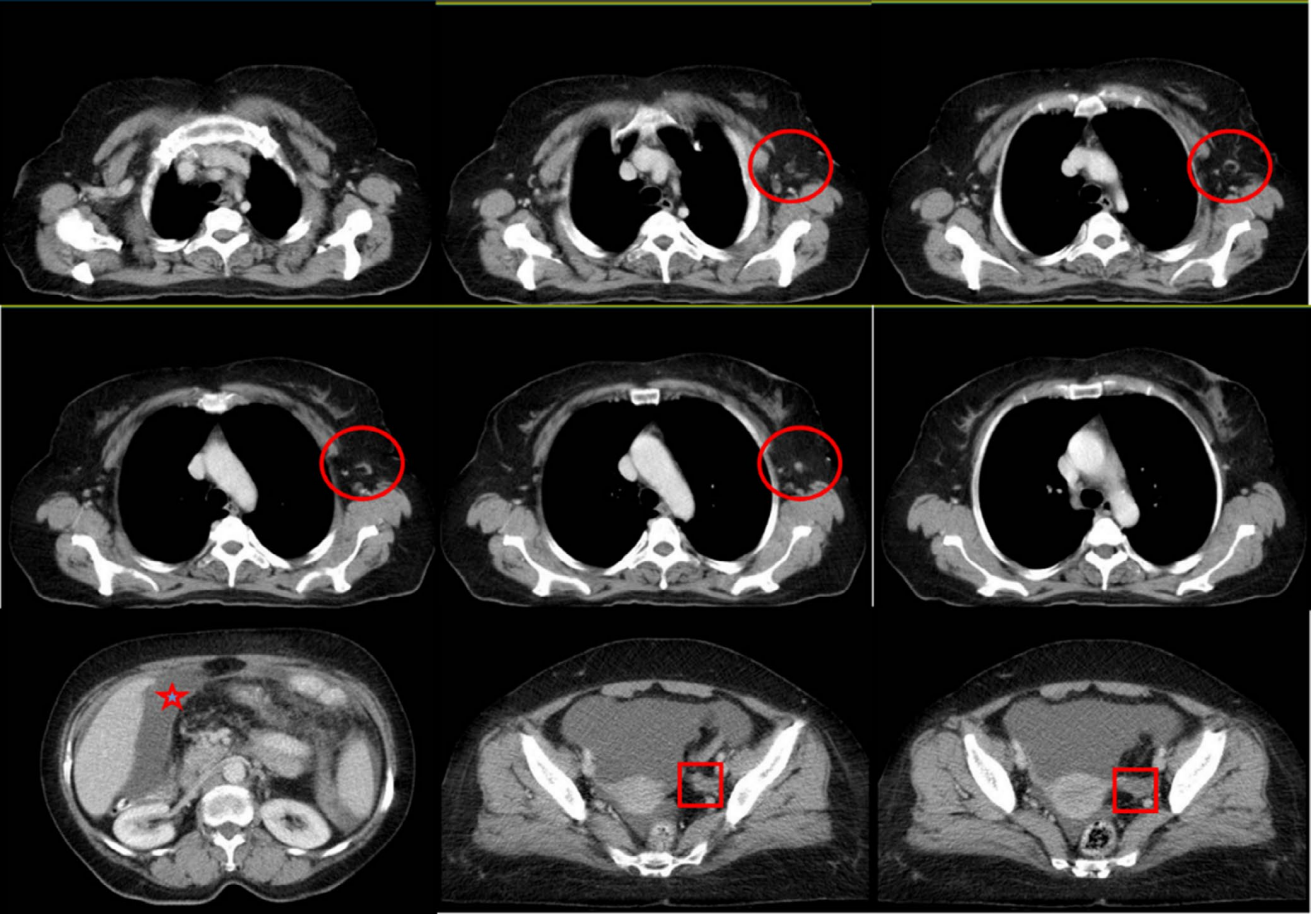
Whole‐body computed tomography (CT) scan showed lymphadenopathies of left axillary region (circle) along with moderate ascites (star) and left ovarian mass (square)

**FIGURE 2:**
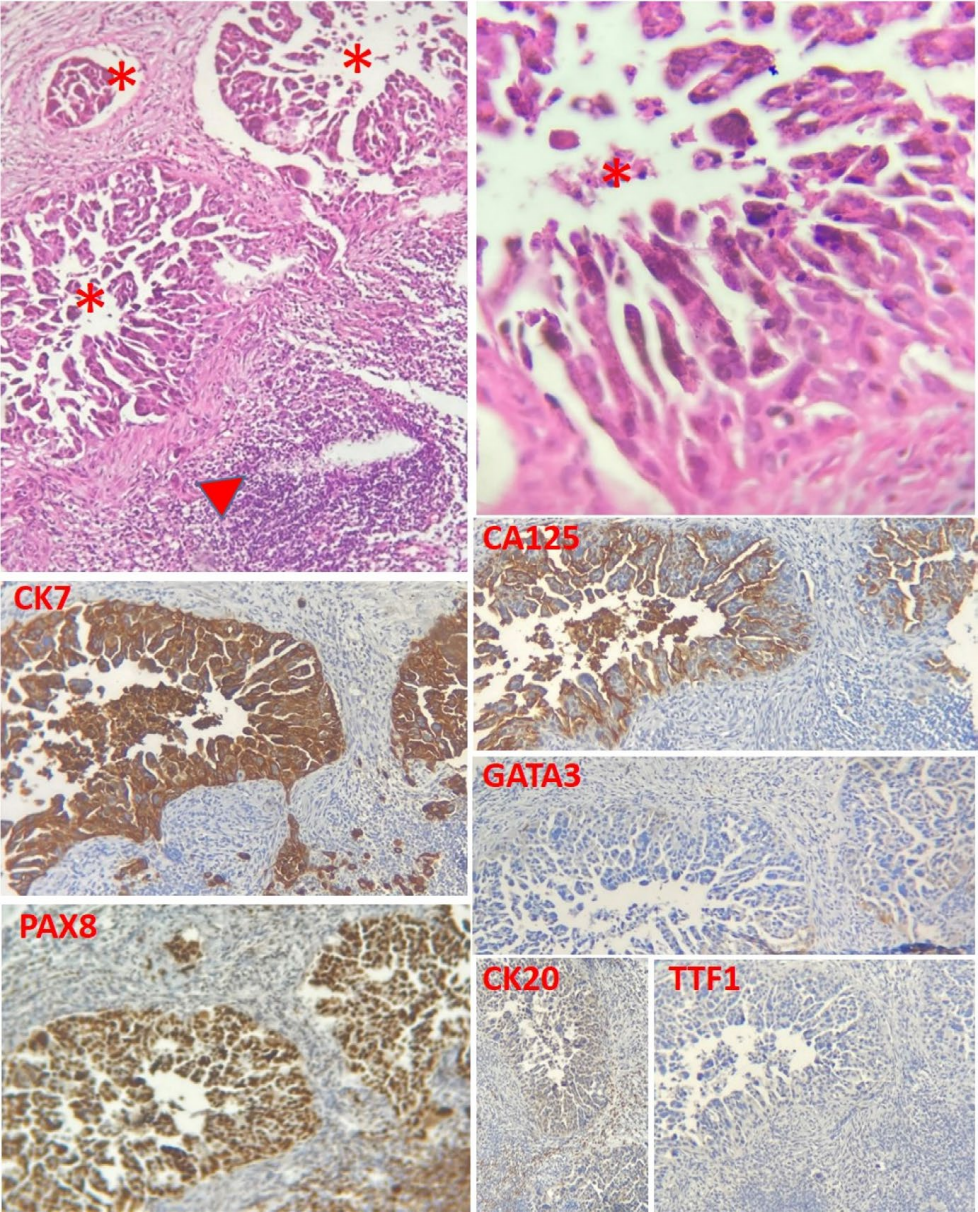
Metastatic carcinoma of ovary to axillary lymph‐node. Top left: lymph‐node tissue (arrow head) infiltrated by tumor tissue (stars) composed of glandular and micropapillary structures of malignant epithelial cells (H&E ×100). Top right: H&E ×400

A 55‐year‐old married woman was referred to our department due to swelling of left axillary region. She had no known medical or familial history. Physical examination revealed isolated axillary lymphadenopathies on the left side, which were fixed to surrounding tissues. An axillary ultrasound showed enlarged conglomerate of several metastatic lymph‐nodes in the left axilla with thickened cortex and the disappeared hilum. A core need biopsy of the nodes showed carcinoma of unknown primary origin. While ordering a whole‐body computed tomography (CT) scan and bilateral mammography, a comprehensive immunohistochemical panel was requested to reveal a primary site of origin. The mammograms were normal; however, the CT scan showed lymphadenopathies of left axillary region along with moderate ascites and left ovarian mass (Figure 1). Morphological examination showed infiltration of tumor tissue (stars) composed of glandular and micropapillary structures of malignant epithelial cells in the lymph‐node and immunohistochemical staining showed a strong cytoplasmic staining for CK7, strong nuclear staining for PAX8, and membrane and luminal staining for CA125, confirming ovarian origin of tumor. Moreover, negative staining of tumoral cells for GATA3, CK20, TTF1 excluded breasts, colon, rectum and lungs as the primary sources of metastasis,  = respectively (Figure 2). A PET/CT scan was requested prior to surgery; however, the patient avoid doing it due to costs. Subsequently, comprehensive biochemistry blood investigations were requested that showed significant increased levels of CA 125 (512 U/ml), while the levels of CA 19–9 and AFP were reported normal.

The patient underwent total abdominal hysterectomy and bilateral salpingo‐oophorectomy (TAH‐BSO) and pathologic examination showed bilateral ovarian high‐grade serous cystadenocarcinoma with FIGO stage IVB (confined to the ovaries). The pathologic assessment specimen resulted from TAH‐BSO showed identical results comparing to the axillary lymph‐node biopsy results. After surgery, four 3‐weekly cycles of adjuvant bevacizumab (15 mg/kg), paclitaxel (175 mg/m^2^), carboplatin (AUC 5) chemotherapy were administered. Then, the patient followed up every 3 months through physical examination and checking serum CA‐125 showing no evidence of axially lymphadenopathy, ascites, and raised tumor marker. Also, a whole‐body CT scan was obtained 1 year after completion of treatments confirming that the patient is disease free. A genetic consultation was requested to assess germline BRCA1/2 mutation and the possibility of PARP inhibitors maintenance therapy. However, patient avoid doing that because of the costs.

Immunohistochemical staining (×100) shows strong cytoplasmic staining for CK7, strong nuclear staining for PAX8, and membrane and luminal staining for CA125, indicating ovarian origin of tumor. The tumor cells are negative for GATA3, CK20, and TTF1, excluding breast, colorectal and lung origin, respectively.

## 3 DISCUSSION

Diagnosis of origin of metastatic diseases when they are presenting in unusual locations and uncommon organs is not hard in patients with active cancers.^10^ However, it might be challenging if it presents as the primary manifestation of malignant disease which is known as carcinoma of unknown primary (CUP). With introduction of gene profiling studies and next‐generation sequencing along with comprehensive, the management of patients with CUP has been evolved during the last decade.^11^

Although with new diagnostic methods such as ^18^F‐fluorodeoxyglucose (FDG) positron emission tomography/computed tomography (PET/CT), the detection rate of non‐reginal lymph‐node metastasis such as supradiaphragmatic lymph‐node involvement may increase.^12^ However, these extensive investigations to detect distant metastasis beyond abdominal cavity have not been recommended, yet.^13^

By diagnosis of metastatic adenocarcinoma involving the axillary lymph‐nodes, our patient considered as favorable CUP.^14^ A primary breast cancer is the most frequent scenario in patients with isolated unilateral axillary lymph‐nodes metastasis.^15^, ^16^ Accordingly, a mammography was requested in our patient which was normal. Moreover, immunohistochemical staining was negative for GATA3, which is a specific marker for breast cancer.^17^ With excluding the breasts as the primary site of malignancy, a whole‐body CT scan was requested which shows moderate ascites and left ovarian mass proposing the ovary as the potential source of metastasis. The primary site assignment of extrauterine high‐grade serous carcinoma requires sectioning and extensively examining the FIMbriated end protocol to rule out serous tubal intraepithelial carcinoma.^18^ Based on our case, both tumoral lesions were confined to the both ovaries.

After the primary TAH‐BSO, bevacizumab, paclitaxel, and carboplatin were administered as the adjuvant treatment and subsequently patient underwent regular follow‐up.^19^ Despite the importance of genetic assessment and its impact on the treatment of patients with cancer,^20^, ^21^, ^22^ the patient denied to perform it due to costs limiting the treatment options.^23^

## 4 CONCLUSION

In the absence of malignant lesion in a breast of patient with CUP limited to axillary lymph‐nodes, ovaries should be considered as a potential source of metastasis.

## CONFLICT OF INTEREST

The authors declare that they have no conflicts of interest.

## AUTHOR CONTRIBUTION

D.F., M.M., and Ab.E. supervised the case report and contributed to the final version of the manuscript. M. Gh., A.A.F., and Az.E. contributed to the interpretation of the results and took the lead in writing the manuscript. S. Sh. provided the pathologic images and contributed to the interpretation of them. All authors discussed the results and commented on the manuscript.

## CONSENT

An informed written consent form was obtained from the patient.

## Data Availability

The data sets used and/or analyzed during the current study are available from the corresponding authors per request.
